# Systems controls are needed to reduce mistaken tests for hemophagocytic lymphohistiocytosis, results of a prospective quality-improvement cohort study

**DOI:** 10.1097/MD.0000000000026509

**Published:** 2021-07-02

**Authors:** Salahuddin Safi, Satish Shanbhag, Bryan C. Hambley, Samuel A. Merrill

**Affiliations:** a West Virginia University School of Medicine, Department of Medicine, Section of Hematology/Oncology, Morgantown, WV; b Consultant Hematologist/Oncologist, Cancer Specialists of North Florida; Adjunct Associate Professor of Medicine, Division of Hematology, Johns Hopkins University, Baltimore, MD; c Department of Internal Medicine, Division of Hematology/Oncology, University of Cincinnati, Cincinnati, OH.

**Keywords:** adults, bone marrow, diagnostic hematology, failure, hemophagocytic lymphohistiocytosis, NK cells

## Abstract

Medical diagnosis and therapy often rely on laboratory testing. We observed mistaken testing in evaluations for hemophagocytic lymphohistiocytosis (HLH) that led to delays and adverse outcomes. Physicians were mistakenly ordering interleukin-2 and quantitative natural killer cell flow cytometry, rather than soluble interleukin 2 receptor (sIL2R) or qualitative natural killer functional tests in the evaluation of patients suspected to have HLH.

We initiated a prospective quality improvement project to reduce mistaken testing, reduce delays in correct testing due to mistaken ordering, and improve HLH evaluations. This consisted of provider education, developing an evaluation algorithm, and ultimately required systems interventions such as pop-ups and removal of the mistaken tests from the electronic ordering catalog.

Active education reduced mistaken testing significantly in HLH evaluations from baseline (73.3% vs 33.3%, *P* = .003, relative risk reduction (RRR) 54.5%), but failed to meet the pre-specified RRR cutoff for success (70%). Education alone did not significantly reduce the proportion of HLH evaluations with delays in sIL2R testing (23.3% vs 7.4%, *P* = .096). Mistaken testing increased after the active intervention ended (33.3% vs 43.5%, *P* = .390, with RRR 40.7% from baseline. Mistaken test removal was successful: mistaken testing dropped to 0% (*P* < .001, RRR 100%), saved $14,235 yearly, eliminated delays in sIL2R testing from mistaken testing (23.3% vs 0%, *P* = .008), and expedited sIL2R testing after admission for HLH symptoms (14.6 days vs 3.8 days, *P* = .0012). These data show systems controls are highly effective in quality improvement while education has moderate efficacy.

## 1 Introduction

The appropriate use of laboratory tests is crucial in modern medicine, especially in hematology due to test abundance and differing methodologies. Inappropriate ordering and/or misinterpretation of such tests can increase healthcare costs, contribute to misdiagnosis or delays in care, and contribute to adverse patient outcomes.^[1–3]^ Unfortunately, inappropriate laboratory testing is common, and occurs due to deficits in knowledge, awareness, and experience.^[4]^ The potential for adverse outcomes is magnified in difficult to diagnose, high acuity conditions such as hemophagocytic lymphohistiocytosis (HLH), where timely diagnosis and treatment are necessary because 30-day mortality is high (21%–27%),^[2,5]^ treatment delay is associated with inferior survival,^[6]^ and mistaken testing can be common.^[2]^

Although educational interventions, such as the Choosing Wisely campaign, help to empower clinicians and patients to challenge appropriate test usage, education alone may benefit from systems controls to prevent or minimize inappropriate test utilization.^[7,8]^ We undertook a prospective quality improvement project in adult HLH because we observed the interleukin-2 (IL2) was being mistaken for soluble IL2 receptor (sIL2R, also known as soluble CD25) testing, and natural killer (NK) number was mistaken for NK function when patients were evaluated for HLH by the HLH-2004 diagnostic criteria.^[9]^ The objectives were to reduce mistaken testing, encourage correct testing, and reduce delays in diagnostic testing arising from mistaken test ordering. We found that educational interventions did reduce mistaken HLH diagnostic testing, but this effect was modest in magnitude, transient in duration, and failed to reach the objective. Reaching the objectives required systems controls to actively prevent the ordering of mistaken tests. We report the findings of our multi-year project here to facilitate other quality improvement endeavors regarding laboratory test utilization in hematology and general medicine.

## 2 Methods

This single-center, prospective quality-improvement project with planned iterative observational cohort retrospective review was modeled on prior endeavors,^[10]^ and general methods were reported previously.^[2]^ The primary aim of this intervention was to reduce mistaken testing in HLH evaluations that could lead to misdiagnosis, delayed diagnosis, or delayed treatment. The secondary aims were to increase correct test ordering (sIL2R), reduce the time from admission to test ordering (sIL2R), and reduce costs from mistaken testing. The Johns Hopkins Hospital is a tertiary referral hospital located in Baltimore, MD, with approximately 1100 inpatient beds for inpatient general medicine and specialty services.

Clinically we observed 2 patterns of repetitive, erroneous HLH diagnostic testing: 1) IL2 was mistaken for sIL2R and 2) NK quantification by in-house or send out (Quest Diagnostics) flow cytometry was confused with NK functional assays that were only available as send out tests. NK functional assays utilized in the diagnosis of HLH may be either NK flow cytometry assays for perforin or CD107a, or in-vitro ^51^Cr release assays,^[11–13]^ but NK quantification is not a recognized diagnostic assay for HLH. NK functional tests are often practically difficult to obtain due to sample volume requirements in the setting of severe cytopenias, sample viability, and shipping logistics for sample transport to the specialized testing center used by our institution (Cincinnati Children's Hospital, Cincinnati, OH). In our electronic ordering system, sIL2R appeared as “‘Interleukin-2 receptor, EIA” and IL2 appeared as “Interleukin-2, circulating.” NK functional assays were not orderable electronically and required a paper lab requisition, however, “Natural killer cells” was a quantitative assay that appeared in the electronic ordering system. Due to this arrangement, the electronic order for NK testing functioned as a decoy, and function testing was rarely ordered by clinicians testing for HLH. “Natural killer cells” was not intended for clinical utilization, but was a legacy of the pathology workflow for flow cytometry analysis. The configuration of the IL2 and sIL2R orders leads to both tests being ordered, or alternatively, the correct test is omitted.

The quality improvement intervention began as an educational effort to increase awareness in hematology fellows and hematology attending physicians by way of hematology conference presentations, case discussions, and in-person consultations. This target group was selected because they provided consultation recommendations to medical teams evaluating patients for HLH, and directed evaluation of patients with HLH on the Hematology inpatient service. A standardized consult note was developed with instructions on correct test ordering, and this incorporated a search strategy for HLH triggers. Additional outreach education was done with the internal medicine intern class during their orientation in July, 2017 with the goal of preventing mistaken test ordering due to differential trainee experience and medical knowledge. An electronic health record clinical decision support tool was in development for identifying HLH triggers and early diagnosis facilitation with the HScore. This mechanism was not advanced at the institutional level due to concerns that it could actually increase inappropriate evaluations and healthcare waste by facilitating HLH testing in patients unlikely to have HLH. An electronic pop-up was utilized to alert clinicians to mistaken test ordering until the mistaken tests could be removed from the ordering interface.

Adult patients (age ≥18) evaluated for possible HLH were identified from administrative and testing databases by 1) International Classification of Diseases (ICD) 9th revision code 288.4, ICD-10 code D76.1X; 2) NK flow cytometry, IL2, or sIL2R testing; and 3) consultation logs for HLH consultation. The charts were reviewed, and clinical information was recorded in a standardized form. HLH was defined by meeting ≥5 of 8 HLH-2004 criteria or HScore ≥169, as detailed previously.^[2,9,14]^ Patients were excluded from analysis if they were not evaluated for HLH, or if they were less than 18 years of age. Testing of IL2 or NK flow cytometry was defined as a mistaken test, and the intent of the testing was verified by medical record review for each occurrence. Test number, order date, test type, treating service, as well as testing delay for intended testing were recorded on a standardized form. Ordering of NK functional tests was not expressly encouraged due to prior expert opinion about limited utility in adult HLH during the intervention period.^[15]^ All evaluations, testing, procedures, and therapeutic decisions were at the discretion of the treating clinicians. HLH genetic testing was obtained with the Cincinnati Children's Hospital HLH panel, and another genetic testing was obtained by the treating clinicians when an inherited syndrome was suspected. Test costs were provided by Quest Diagnostics. None of the participants had any financial conflicts of interest in the intervention. Patient privacy was protected in agreement with standard hospital policies. The iterative retrospective cohort reviews were conducted after approval of the Johns Hopkins Institutional Review Board. Bias was minimized by including patients from problem list billing databases as well as consultations, not just test result databases, as well as pre-specified endpoints, and objective measures of delay. Although the quality improvement intervention could not reach all providers at the institution simultaneously, recurring presentations were done to increase the exposure of the target audience.

Pre-specified time periods for analysis were January, 2014 to December, 2015 pre-intervention baseline; January, 2016 to August, 2016 education intervention (when active education was used); September, 2016 to October, 2017 education washout (active education stopped, but all prior resources were available); October, 2017 to April, 2018 test elimination 1 (starting when IL2 test was removed from electronic test catalog); and May, 2018 to February, 2019 test elimination 2 (when NK testing was removed from the electronic test catalog). Patients were followed until June 2019 when the project closed. Education intervention success was pre-specified and defined as 70% or greater relative reduction in mistaken test ordering, with complete elimination as the intended goal of the project. Interim analysis of education success was done in January, 2017 with retrospective evaluation of the cohort.

### 2.1 Statistical methods

Descriptive statistics were computed with percentages, frequencies, medians, and means as indicated. Relative risk reduction was defined as 1 − (proportion with mistaken testing in the intervention group)/(proportion with mistaken testing at pre-intervention baseline). Overall survival was defined as the date of initial hospitalization at our institution for HLH symptoms until death. Delay was defined as the number of days between mistaken test ordering and appropriate test ordering. To control confounding from variable test result turnaround times, the test ordering date was used. Patients missing testing data were not included in analyses of delays. Associations between mistaken testing and medical service were analyzed. Assuming a priori that 2 HLH evaluations per month would occur with a minimal 50% intervention effect size, 0.95 confidence level, and 0.05 confidence interval, our target intervention sample size was 15 patients for each quality improvement intervention period. Kaplan–Meier survival analysis and the log-rank test were used to compare survival where indicated. Quality improvement outcomes were analyzed with the test of proportions or unpaired *t* test for continuous variables as indicated. All tests were 2-sided and performed at a 0.05 level of statistical significance. Computations utilized STATA16, (StataCorp. 2019. Stata Statistical Software: Release 16. College Station, TX).

## 3 Results

During the study period, 170 potentially eligible patients were identified; 13 were excluded due to age <18, and 9 adults were excluded due to not being evaluated for HLH. From this, 148 adults were evaluated for HLH during the study period (Table 1). Of patients evaluated for HLH, 62.2% and 77.0% met HLH-2004 and HScore criteria for HLH, respectively. Relevant to the quality improvement project, 7.4% of all evaluations did not have sIL2R testing and 96.6% did not have NK functional testing. Reporting HLH etiologies and treatments is outside the scope of this current report and has been reported in other series. In those fulfilling HLH-2004 diagnostic criteria who had genetic testing, the majority had negative HLH gene panel testing (66.7%); however, we found that immunodeficiency (CTLA4 deficiency, RAS-associated lymphoproliferative disorder) was as common as mutations traditionally associated with HLH (LYST, XIAP). An insufficient sample for genetic testing was noted in 11.1% of those with the testing ordered.

**Table 1:** Patient characteristics of HLH evaluations.

Criteria, unit	No.	%	Median	Range
Age, years	148/148		54	19–81
Sex, male	83/148	56.1%		
Race				
White	71/148	48.0%		
Black	59/148	39.9%		
Other	18/148	12.2%		
HScore ≥169^∗^	114/148	77.0%	221	33–322
≥5 HLH-2004 criteria	92/148	62.2%	5	1–7
Ferritin, ≥500 ng/mL^∗^	135/139	97.1%	10499	12–326,604
Hemoglobin, <9 g/dL^∗^	131/148	88.5%	6.6	3.6–12.8
Platelet, <100 × 10^9^/L^∗^	121/148	81.8%	33	0–347
Fever, ≥38.5°C	119/148	80.4%	39.3	34.4–42.9
sIL2R, ≥2400 U/mL	105/137	76.6%	5018.5	406–151941
Splenomegaly	82/142	57.7%		
Prior splenectomy	3/148	2.0%		
Triglyceride, ≥265 mg/dL^∗^	64/143	44.8%	236	48–994
Hemophagocytosis^†^	46/103	44.7%		
ANC, <1 × 10^9^/L^∗^	50/148	33.8%	1630	0–48000
Fibrinogen, ≤150 mg/dL^∗^	35/138	25.4%	272.5	35–1290
NK function	5/148	3.4%		
Insufficient sample	2/5	40.0%		
Absent or decreased	3/5	60.0%		
Genetic testing	18/148	12.2%		
CTLA4	1/18	5.6%		
XIAP	1/18	5.6%		
RAS	1/18	5.6%		
LYST	1/18	5.6%		
Negative	12/18	66.7%		
Insufficient sample	2/18	11.1%		

ANC = absolute neutrophil count, HLH = hemophagocytic lymphohistiocytosis, NK cell = natural killer cell.

∗ Within 7 days of hospital admission.

† On bone marrow biopsy, lymph node biopsy, or liver biopsy.

In the pre-intervention baseline period, 30 patients were evaluated for HLH (Table 2). The majority, 73.3% of patients evaluated, had mistaken HLH testing. Mistaken testing led to excess costs of $10,963.39 or $5,481.50 per year, which was $365.45 per patient evaluated. Mistaken IL2 testing led to delays in ordering sIL2R testing in 23.3% of all patients evaluated for HLH in the pre-intervention period, and the mean delay was 9.1 days. During the active education intervention period, 27 patients were evaluated for HLH. Mistaken testing was observed in 33.3% of patients, and led to $3155 in excess costs, or $116.87 per patient evaluated. Mistaken IL2 testing led to sIL2R delays in 7.4% of patients, and the mean delay of 10 days in the education period. There was no significant difference between the proportion of patients with sIL2R delays (*P* = .096) between pre-intervention and active education groups. Although the intervention reduced the proportion of patients with mistaken testing (*P* = .003), the relative risk reduction of mistaken testing was 54.5% and thus failed to meet the pre-specified goal of 70%.

**Table 2:** Different interventions and their effects on evaluation of HLH.

	Intervention period				
Outcome	Pre-intervention^∗^	Active education^†^	Washout^‡^	Eliminate 1^§^	Eliminate 2^||^
No mistaken testing, n (%)	8/30 (26.7)	18/27 (66.7)	26/46 (56.5)	15/18 (83.3)	27/27 (100)
Mistaken testing, n (%)	22/30 (73.3)	9/27 (33.3)	20/46 (43.5)	3/18 (16.7)	0/27 (0)
NK testing	20/22	8/9	13/20	3/3	0
IL2 testing	10/22	3/9	9/20	0/3	0
sIL2R delay from mistaken testing, n (%)	7/30 (23.3)	2/27 (7.4)	2/46 (4.3)	0/18 (0)	0/27 (0)
mean delay, days (range)	9.1 (1–24)	10.0 (8–12)	1.5 (1–2)	N/A	N/A
sIL2R not obtained	1/30 (3.3)	0/27 (0)	4/46 (8.7)	3/18 (16.7)	2/27 (7.4)
IL2 tests ordered	11	3	9	0	0
Cost	$1565.74	$427.02	$1281.06	$0.00	$0.00
NK tests ordered	31	9	16	4	0
Cost	$9397.65	$2728.35	$4850.40	$1212.60	$0.00
Total mistaken test cost	$10963.39	$3155.37	$6131.46	$1212.60	$0.00
Cost of mistaken testing/patient evaluated	$365.45	$116.87	$133.29	$67.37	$0.00
Proportion with mistaken testing, *P* value	Reference	.003	.017	<.001	<.001
Proportion with mistaken testing, *P* value	−	Reference	.390	.217	.001
Mistaken testing relative risk reduction	Reference	54.5%	40.7%	77.3%	100.0%
Proportion with sIL2R delay, *P* value	Reference	.096	.013	.028	.008
Proportion with sIL2R failure, *P* value	Reference	.341	.353	.103	.488
Proportion with sIL2R failure, *P* value	−	Reference	.115	.028	.150

HLH = hemophagocytic lymphohistiocytosis, IL2 = interleukin-2, NK cell = natural killer cell, sIL2R = soluble interleukin 2 receptor.

∗ Pre-intervention period: Period before active education and elimination of inappropriate testing (to assess baseline practices).

† Active education: period where the providers were educated about appropriate use of the tests for HLH (to assess effects of active education on ordering appropriate testing.

‡ In washout period active education was stopped (but all prior resources used in active education were still available to be used).

§ Eliminate 1: Period starting after IL2 test was removed from electronic test catalog.

|| Eliminate 2: Period starting after NK testing was removed from the electronic test catalog.

**Figure 1:**
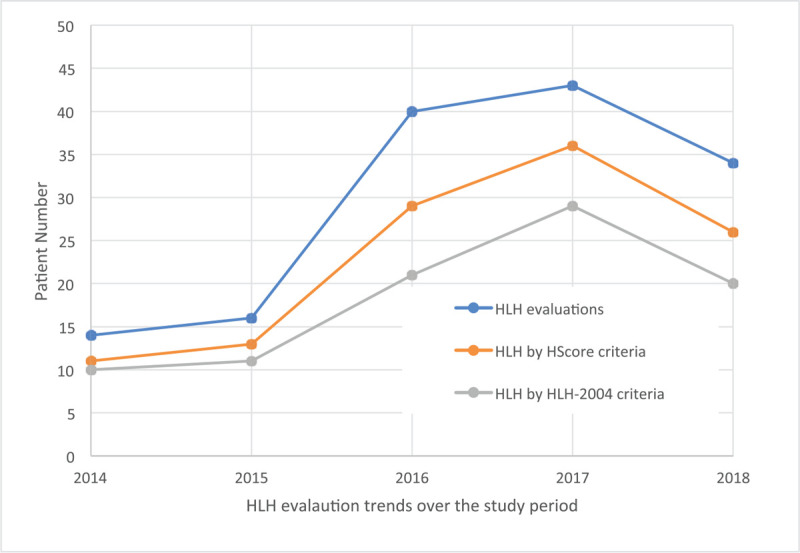
Trends in HLH evaluations over time. HLH evaluations by year are indicated in the circles. HLH cases as defined by meeting HScore criteria are shown in squares. HLH cases defined as meeting HLH-2004 criteria are shown in triangles. Trend toward the increase of HLH evaluations during the study period. HLH = hemophagocytic lymphohistiocytosis.

During the active education and washout periods, alternative methods to reduce mistaken testing were explored in discussions with pathology and laboratory medicine. A decision support tool and HLH order set were ultimately not feasible due to concerns of increased test utilization from facilitating extensive testing in greater numbers of patients. This concern was validated by the observed increase of HLH evaluations during the study period (Fig. 1). Review of all IL2 and NK testing at the institution in the pre-intervention period revealed that testing outside of HLH evaluation (n = 2) was not useful. Since IL2 testing did not serve the clinical purpose and the NK cell order was not intended to be an orderable entity, test removal was recommended but would take considerable time to achieve. An electronic pop-up was developed during the washout period as a temporary measure; this deployed when IL2 or NK testing was ordered and alerted the clinician that these were not tests for HLH and to contact hematology for guidance. Ultimately IL2 and NK flow cytometry were removed from the electronic test catalog after demonstrating lack of utility of the tests, high frequency of mistaken ordering, impacts of delayed diagnosis, and increased costs.

In the washout period, when the active educational effort had ended, mistaken testing increased from 33.3% to 43.5% of HLH evaluations, but this was not statistically significant (*P* = .390). Costs of mistaken testing increased from $116 to $133 per patient evaluated. Removal of IL2 (elimination period 1) and NK testing (elimination period 2) from the electronic test catalog were successful in abolishing mistaken testing (0%, *P* < .001), met the pre-specified 70% reduction for mistaken testing, and significantly reduced the proportion of patients with delays in obtaining sIL2R testing (from 73.3% in pre-intervention vs 0% after elimination, *P* < .001). With an average of 39 HLH evaluations per year between 2016 and 2018, and costs of $365 per patient on mistaken testing before the intervention, test elimination was projected to save $14,235 yearly from inpatient diagnostic costs based on pre-intervention testing frequencies. Time from patient hospital admission to ordering sIL2R testing for HLH evaluation decreased from a mean of 14.6 days in the pre-intervention period to 3.8 days in elimination period 2 (*P* = .0012).

Although most patients were evaluated for HLH on the medical intensive care (28.4%,) general internal medicine (23.6%), or hematologic oncology services (23.6%), several other subspecialty services were conducted HLH evaluations in adult patients (Table 3). Mistaken HLH testing was obtained by multiple services, but there were no significant differences between the proportions of HLH evaluations with mistaken testing by primary medical service. Consistent with the premise that IL2 is not related to HLH, the sensitivity of elevated IL2 for HLH was 0.06 (Table 4); although the only patient with an abnormal IL2 (39 pg/mL, normal range <38 pg/mL) had HLH and thus the specificity was 1.0, this would not support IL2 testing as a diagnostic criterion of HLH. Likewise, NK number below the normal range (normal range 7%–31%) was poorly sensitive for HLH (0.50), and specificity was 0.71. Patients with missing IL2 or NK results were excluded from sensitivity and specificity calculations (IL2 = 1, NK = 3 with HLH, 3 without HLH); all missing results were due to sample collection or analysis problems. Overall survival for patients with HLH by HLH 2004 criteria were not significantly changed during the study (log-rank *P* = .287).

**Table 3:** Mistaken testing based on ordering service evaluating For HLH.

					Mistaken testing					
	All evaluations		Pre-intervention		All evaluations			Pre-intervention		
Evaluating primary service	No.	%	No.	%	No.	%	*P* value	No.	%	*P* value
										
Medical ICU	42/148	28.4%	18/30	60.0%	20/42	47.6%	.432	14/18	77.8%	.904
General internal medicine	35/148	23.6%	3/30	10.0%	10/35	28.6%	.536	1/3	33.3%	.270
Hematologic oncology	35/148	23.6%	3/30	10.0%	9/35	25.7%	.394	2/3	66.7%	.810
Benign hematology	19/148	12.8%	4/30	13.3%	7/19	36.8%	Ref.	3/4	75.0%	Ref.
Infectious diseases	5/148	3.4%	1/30	3.3%	3/5	60.0%	.349	1/1	100.0%	.576
Pediatrics	4/148	2.7%	1/30	3.3%	2/4	50.0%	.623	1/1	100.0%	.576
Transplant Hepatology	3/148	2.0%			0/3	0.0%	.203			
Cardiology	3/148	2.0%			2/3	66.7%	.323			
Cardiac surgery	1/148	0.7%			1/1	100.0%	.201			
Gastroenterology	1/148	0.7%			0/1	0.0%	.452			

HLH = hemophagocytic lymphohistiocytosis, ICU = intensive care unit.

**Table 4:** IL-2 and NK-cell quantification and HLH diagnostic utility.

IL2 test	HLH	Not HLH	NK quantification	HLH	Not HLH
Test positive	1	0	Test positive	16	2
Test negative	16	5	Test negative	16	5
Sensitivity^∗^	0.06		Sensitivity^∗∗^	0.50	
Specificity	1.00		Specificity	0.71	
PPV	1.00		PPV	0.89	
NPV	0.24		NPV	0.24	

HLH = hemophagocytic lymphohistiocytosis, IL2 = interleukin-2, NK cell = natural killer cell, NPV = negative predictive value, PPV = positive predictive value.

∗ Sensitivity of IL-2 Level to diagnose HLH is 6%.

∗∗ Sensitivity of NK cell number to diagnose HLH is 50%.

## 4 Discussion

Diagnostic testing for HLH utilizes laboratory methods that may be unfamiliar to general internal medicine physicians and practicing hematologists. Omission of intended testing or misinterpretation of incorrect tests can have serious implications for patient care, contribute to diagnostic delay, misdiagnosis, and adverse outcomes. Here, we report the methods and results of a more than 3 years long prospective quality improvement project to reduce mistaken testing in patients evaluated for HLH. The initial education effort was time and effort-intensive, and while it reduced mistaken testing, it failed to meet the pre-specified criteria for success. Ultimately, removal of the mistaken tests from the electronic ordering catalog was necessary to achieve the primary and secondary objectives. Test removal decreased costs, decreased sIL2R testing delays, and did not negatively affect other evaluations. The processes and negotiations to remove the mistaken testing were much more onerous than initially anticipated because of the burden of proof required for test removal from the electronic catalog, accounting for the long duration of a project that is conceptually simple.

Other reports suggest that mistaken testing in HLH was not limited to our institution,^[16,17]^ however, the extent of mistaken testing at other centers is not known. We found that IL2 and NK numbers were not helpful for HLH diagnosis. In recent prospective trial adults with HLH were found to have impaired NK function,^[18]^ contrary to prior expert opinion when this project was initiated, however, we observed this testing was seldom obtained due to mistaken ordering, and sample collection problems when the correct test was ordered. The methodological differences between NK functional testing, along with a similarly named decoy test easily available in our electronic ordering system, contributed to the common practice of clinicians ordering and then misinterpreting a mistaken test. Similar cognitive bias was observed with IL2 and sIL2R (also known as sCD25a), despite both tests being in the electronic ordering system.

Timely treatment of HLH can improve patient outcomes, and sIL2R can aid in HLH diagnosis and provide prognostic information.^[6,19,20]^ This project began after mistaken NK testing led to an inaccurate exclusion of HLH as the cause of a patient's illness, only to have the patient present at another hospital shortly after with fulminant HLH. Review of mistaken testing by ordering service revealed that the mistaken testing was not specific to 1 primary medical team, but was a systematic practice. Anecdotally, the treating medical teams were ordering testing as recommended verbatim by hematology, also not realizing it was incorrect. The sub-optimal effect of education is not entirely surprising in a large academic medical center with frequent staff turnover; however, information dissemination was not the only barrier. For example, 1 author (SAM) was asked by another hematology attending who had attended the prior educational sessions as to why the NK testing for HLH had disappeared from the electronic ordering system, after more than 2 years of education efforts about the topic. These data show that clear and factually accurate recommendation necessary during the consultation but ordering system reform is also needed to minimize mistaken testing.

This prospective quality improvement project has several limitations. First, some centers may not experience this mistaken testing for HLH due to the configuration of their electronic health record, yet we also noticed this testing pattern at our current institution. Inappropriate hematology testing is common and the lessons of this study can apply to other projects, such as hypercoagulable testing in stroke, where mistaken test interpretation is common, can contribute to harm, and is costly.^[3,21]^ Second, the educational intervention was difficult and labor-intensive, and the pop-up intended as a temporary measure was likely, not helpful due to “pop-up fatigue.” While education did have an effect on reducing mistaken testing, its sustainability would have been impossible as enacted. Third, because of the heterogeneity of HLH it was not possible to show that the project led to more rapid HLH diagnosis, specific treatment, or survival advantage. Many patients were correctly treated empirically for their HLH trigger before HLH was considered or diagnosed, such as broad-spectrum antimicrobials or corticosteroids (data not shown). Finally, cost savings of the project ($14,235 yearly) are proportionally small in comparison to other areas in need of hematologic testing stewardship, such as inappropriate hypercoagulable testing that may save more than $100,000 yearly per hospital system.^[3,21–23]^

Successful HLH therapy requires prompt HLH diagnosis and trigger identification. We utilized an iterative quality improvement framework to eliminate mistaken diagnostic testing in HLH evaluations after initial measures were insufficient. While education was helpful, systems controls were required to achieve the objective. Alterations to the electronic test catalog can successfully reduce inadvertent or mistaken test ordering. Use of systems controls in medicine can facilitate other diverse quality improvement efforts, and this experience can help guide clinicians to techniques that can improve care in their area of practice.

## Acknowledgments

The authors acknowledge assistance for clinical data coordination and statistical design from the Center for Clinical Data Analytics, supported in part by the Johns Hopkins Institute for Clinical and Translational Research (ICTR). We thank Ximin Li from the ICTR for statistical consultation during the study.

## Author contributions

**Conceptualization:** Satish Shanbhag, Brian C. Hambley, Samuel A. Merrill, Salahuddin Safi.

**Data curation:** Satish Shanbhag, Brian C. Hambley, Samuel A. Merrill, Salahuddin Safi.

**Formal analysis:** Satish Shanbhag, Brian C. Hambley, Samuel A. Merrill, Salahuddin Safi.

**Investigation:** Satish Shanbhag, Brian C. Hambley, Samuel A. Merrill, Salahuddin Safi.

**Methodology:** Satish Shanbhag, Brian C. Hambley, Samuel A. Merrill, Salahuddin Safi.

**Project administration:** Satish Shanbhag, Brian C. Hambley, Samuel A. Merrill, Salahuddin Safi.

**Resources:** Satish Shanbhag, Brian C. Hambley, Samuel A. Merrill, Salahuddin Safi.

**Software:** Satish Shanbhag, Brian C. Hambley, Samuel A. Merrill, Salahuddin Safi.

**Supervision:** Satish Shanbhag, Brian C. Hambley, Samuel A. Merrill, Salahuddin Safi.

**Validation:** Satish Shanbhag, Brian C. Hambley, Samuel A. Merrill, Salahuddin Safi.

**Visualization:** Satish Shanbhag, Brian C. Hambley, Samuel A. Merrill, Salahuddin Safi.

**Writing – original draft:** Satish Shanbhag, Brian C. Hambley, Samuel A. Merrill, Salahuddin Safi.

**Writing – review & editing:** Satish Shanbhag, Brian C. Hambley, Samuel A. Merrill, Salahuddin Safi.
